# Quantitative assay for the detection of the V617F variant in the Janus kinase 2 (JAK2) gene using the Luminex xMAP technology

**DOI:** 10.1186/1471-2350-11-54

**Published:** 2010-04-01

**Authors:** François W Paradis, Raynald Simard, Daniel Gaudet

**Affiliations:** 1 ECOGENE-21, department of médecine, Université de Montréal, Chicoutimi Hospital, 225 rue St-Vallier, Saguenay, Province of Québec, G7H 7P2, Canada

## Abstract

**Background:**

The availability of clinically valid biomarkers contribute to improve the diagnosis and clinical management of diseases. A valine-to-phenylalanine substitution at position 617 (V617F) in the Janus kinase 2 (JAK2) gene has been recently associated with key signaling abnormalities in the transduction of haemopoietic growth-factor receptors and is now considered as a useful clinical marker of myeloproliferative neoplasms. Several methods have recently been reported to detect the JAK2 V617F point mutation and show variable sensitivity.

**Methods:**

Using the Luminex xMAP technology, we developed a quantitative assay to detect the JAK2V617F variant. The method was based on polymerase chain reaction (PCR) followed by hybridization to specific probes coupled with internally dyed microspheres. The assay comprises 3 steps: genomic DNA extraction, end point PCR reaction, direct hybridization of PCR fragments and quantification. It has been tested with different sources of nucleic acid.

**Results:**

Applied to whole blood samples, this quantitative assay showed a limit of detection of 2%. A highly sensitive allele-specific primer extension reaction performed in parallel allowed to validate the results and to identify the specimens with values below 2%.

**Conclusion:**

Direct hybridization assay using the Luminex xMAP technology allows sensitive quantification of JAK2V617F from blood spots. It is simple and can be easily performed in a clinical setting.

## Background

Clonal dysregulation linked to myeloproliferative neoplasms (MPN) arising from acquired mutations in the haematopoietic progenitor cells lead to a wide range of clonal haematological malignant diseases including polycythemia vera (PV), essential thrombocythemia (ET), myeloid metaplasia with myelofibrosis (MMM), chronic myelomonocytic leukemia (CMML), chronic myelogenous leukemia (CML), hypereosinophilic syndrome (HES), and systemic mast cell disease (SMCD)[1]. These disorders have been studied at the molecular levels and several of them have been associated with gene mutations resulting in constitutive activation of protein tyrosine kinases [2-4].

Jun activated kinase 2 (JAK2) is a cytoplasmic tyrosine kinase with a key role in signal transduction from multiple haemopoietic growth-factor receptors [5]. JAK2 is activated upon the binding of type 1 cytokine ligands including erythropoietin (EPO), granulocyte macrophage-colony stimulating factor (GM-CSF), and thrombopoietin (TPO) with its receptor. This results in the production of red blood cells, granulocytes/macrophages, and platelets, respectively. Recently, several groups have reported a somatically acquired c.1849G>T point mutation in exon 14 of JAK2 gene (JAK2V617F) [6]. This point mutation results in a Valine to Phenylalanine change at position 617 in the JH2 pseudo-kinase domain and may potentially be responsible for key signaling abnormalities observed in several MPN, including PV, ET and IMF [1,7-9]. This mutation generates a constitutively active tyrosine kinase that confers growth factor independence through the loss of autoinhibition leading to a constitutive activation and uncontrolled proliferation of haematopoietic cells [8]. The JAK2V617F variant is associated with increase hemoglobin concentration, increase levels of white blood cells, splenomegaly, and increase risk of leukemic transformation. Although detected at very low levels in healthy donors [10], the reported prevalence of the JAK2V617F mutation in MPN is elevated: 90% in PV, 50% in ET or IMF and less then 20% in atypical CML [6]. Complications linked to MPN include fibrosis, hemorrhage, and thrombosis.

Several methods of detection with a wide range of sensitivity have been published for the quantification of this mutation. These methods include: direct sequencing (DS) or RFLP, pyrosequencing, allele-specific primer extension (ASPE), amplification-refractory mutation sequencing (ARMS), quantitative real time PCR (QRT-PCR) and DNA melting curve analysis [6,11-14]. The Luminex xMAP technology allows simultaneous detection of up to 100 different analytes in a single reaction vessel [15]. This technique is based on PCR reactions followed by direct hybridization (DH) to probes coupled to internally dyed microspheres. Several clinical applications of this flow cytometry-based genotyping technique have been developed and are used for the detection of human pathogens, HLA typing, multiplex screening of genetic diseases, gene expression profiling, risk assessment, diagnosis or clinical follow-up. In this study, we describe a simple quantitative estimation of the JAK2V617F variant applied to whole blood spots stored on FTA cards with a limit of detection of 2%.

## Methods

### DNA extraction

This project is part of a "health technology development and assessment" program in molecular biology conducted with the authorization and support of the Chicoutimi hospital and regional health authorities. Genomic DNA was isolated from 20 mL of cultured HEL 92.1.7 cells (ATCC # TIB-180) grown in RPMI complete growth medium using the QIAGEN blood and cell culture DNA kit 100/G (QIAGEN Inc. Canada). DNA concentration was measured by absorbance at 260 and 280 nm using a spectrophotometer. CEPH (Centre d'Étude du Polymorphisme Humain) DNA was purchased from Coriell Institute (Camden, New Jersey 08103, USA). Genomic DNA was isolated from 2 mm punches of whole blood specimens stored at room temperature on Whatman's FTA paper and processed as previously described [16]. Briefly, a 2 mm punch was submerged in 100 μL of methanol and allowed to evaporate overnight. The next day, 25 μL of sterile water was added and the solution incubated at 99°C for 10 min. Once cooled at room temperature, the heating step was repeated and then the tubes were spun at 3000 × g for 5 minutes. The supernatant containing the genomic DNA was transferred to a clean tube and further used for amplification of the targeted sequence using an end point PCR as well as an allele-specific primer extension (ASPE) reaction.

### Endpoint PCR and allele-specific primer extension reactions

The end point PCR reactions were carried out using 2.5 μL of FTA purified genomic DNA in a final volume of 25 μL containing: 1× NEB reaction buffer, 250 μM dNTPs, 0.3 μM of each PCR primers (Table 1) and 1 unit of Taq DNA polymerase (New England Biolab, Canada). The allele-specific primer extension (ASPE) reactions were performed using a forward mutant-specific primer, a 3' phosphate-blocked forward wild-type specific primer and a reverse primer (Table 1). The amplification conditions for both types of reactions were as follow: initial denaturation at 95°C for 10 min followed by 45 cycles of denaturation at 95°C for 30 sec, annealing at 60°C for 1 min and elongation at 72°C for 1 min. A final step at 72°C for 5 min was added and samples were kept at 4°C until used. All PCR reactions were performed on a Tgradient thermocycleur (Montreal Biotech Inc, Canada). Amplicons were visualized from a 2.0% agarose-gel electrophoresis under standard conditions. DNA was quantified using the Quant-iT Picogreen dsDNA reagent as described by the manufacturer (Invitrogen, cat. # P11496). The oligonucleotide primers were purchased from Integrated DNA Technology Inc. (Coralville, IA). Negative controls using sterile water were systematically included in all amplification reactions.

**Table 1:** Primers and probes used in end point PCR, ASPE and DH reactions.

PCR	Forward primer	5'-AGCAAGCTTTCTCACAAGCA-3'
	Reverse primer	5'biotin-CTGACACCTAGCTGTGATCCTG-3'
DH	Wild-type probe	5'unilinker-TGGAGTATGT**G**TCTGTGGAGA-3'
	Mutant probe	5'unilinker-TGGAGTATGT**T**TCTGTGGAGA-3'
ASPE	Forward primers	5'-AGCATTTGGTTTTAAATTATGGAGTATATT-3'
		5'-AGCATTTGGTTTTAAATTATGGAGTATGTG-3'PO4
	Reverse primer	5'biotin-CTGACACCTAGCTGTGATCCTG-3'

The reverse primer is biotinylated at its 5' end. The probes differing by a single nucleotide (bold letter) contain a unilinker at their 5' ends for coupling to the microspheres.

### Coupling the oligonucleotide probes to the microspheres and optimization of the hybridization assays

The wild-type probe was coupled to the carboxylated microspheres (xMAP; Luminex Corp., Austin, TX) bead number 064 (Luminex product number L100-C164-01) and mutated probe (Table 1) was coupled to the carboxylated microspheres bead number 065 (Luminex product number L100-C165-01) as described [17]. Each hybridization reaction comprised 2.5 μL of a 1 in 5 diluted PCR products, 10 μL of TE buffer and 25 μL of 1.5× TMAC buffer containing approximately 1500 of each microspheres. The mixture was incubated at 95°C for 10 minutes followed by incubation at 53°C for 30 minutes. To pellet the microspheres, the reactions were quickly spun at 3000 × g for 5 min at room temperature. The supernatant was then removed and the microspheres resuspended in 75 μL of 1× TMAC hybridization buffer containing 4 μg/mL streptavidin-R-phycoerythrin (Invitrogen cat. # S866). The plate was kept in the dark for 10 min and the beads were analyzed at room temperature for assessment of the internal bead color and R-phycoerythrin reporter fluorescence on a Luminex 100 analyzer with the following settings: 45 seconds-50 μL-100 beads. The median fluorescence intensity (MFI) of at least 100 beads was computed for each bead set in the sample. The probes with a unilinker at the 5'ends were purchased from Integrated DNA Technology Inc. (Coralville, IA). The optimal temperature of hybridization was estimated by performing the hybridization at temperatures ranging from 40°C up to 70°C. The effect of DNA concentration on the kinetic of the hybridization reaction was evaluated by testing 5 μL of the PCR products followed by dilutions down to 1:128 and hybridizing at 53°C. The effect of time on the kinetic of the hybridization reaction was determined by using 2.5 μL of PCR product and incubating at 53°C from 0 to 60 min.

### Standard curve and quantitative detection of JAK2

The standard curves were generated using the wild-type allele (CEPH DNA) PCR product and the mutated allele (HEL DNA) PCR product diluted five fold and combined together in order to generate a 0 to 100% range of the wild-type to mutated allelic ratios. For the quantitative detection of JAK2V617F, the specimens PCR products were also diluted five fold and then hybridized to the coupled microspheres as described above. The difference between wild type and mutant probes median fluorescence intensity values were used to generate the standard curve and specimen's profiles.

## Results

### Specimens, Primers and PCR

The initial step of our procedure was to choose the type of genomic DNA and the DNA purification procedure for our assay. To ensure confidentiality of the donor, an aliquot of 50 μL of fresh blood collected in EDTA-containing vaccutainer tubes was spotted onto an FTA card and identified with a number prior to drying overnight. A single FTA card could contain up to 5 specimens linked to its code bar and was easily stored at room temperature in the dark. A single 2 mm punch was sufficient to provide enough genomic DNA for at least 8 genotyping assays. An additional boiling step was added to the original procedure to improve the efficiency of genomic DNA recovery. The amplification of the JAK2V617F targeted region produced a single band of the expected size (155 bp) as visualized from 2% agarose-gel electrophoresis stained with ethidium bromide (not shown). Primers were selected in regions with no known genetic variants preventing the possibility of an allele drop out phenomenon during amplification of the targeted region. To reach the end point status, 45 cycles proved to be sufficient and safe enough assuming 100% amplification efficiency for each PCR cycle. All reactions reached the end point using the procedure described above. Since the primers represent the limiting factor in our PCR reactions, the amount of DNA fragments generated during PCR was about 800 ng which is in agreement with the amount of primers used.

### Determination of the optimal kinetic parameters for direct hybridization

**Figure 1:**
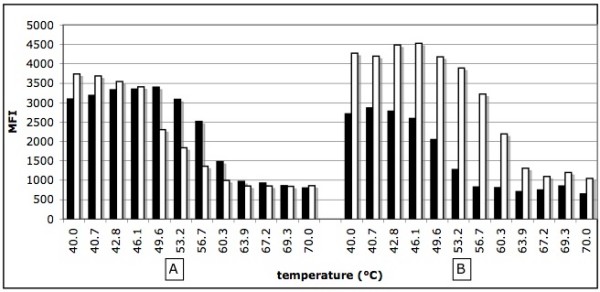
**Effect of temperature on the kinetic of hybridization**. Panel A; homozygous wild type DNA, panel B: homozygous mutated DNA. Black bars correspond to the wild type probe (V617Fwt) and white bars correspond to mutated probe (V617Fmut). MFI: median fluorescent intensity.

**Figure 2:**
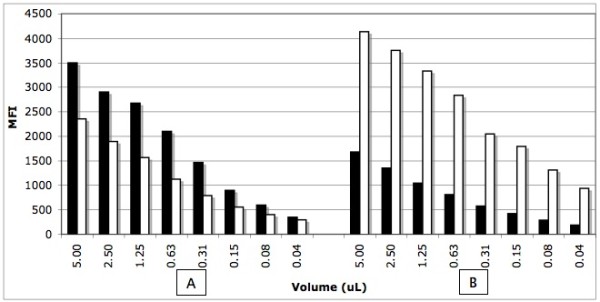
**Effect of the DNA concentration of the kinetic of hybridization**. Panel A; homozygous wild type DNA, panel B: homozygous mutated DNA. Black bars correspond to the wild type probe (V617Fwt) and white bars correspond to mutated probe (V617Fmut). MFI: median fluorescent intensity.

**Figure 3:**
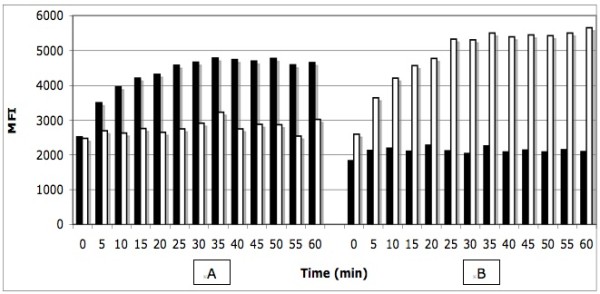
**Effect of time on the kinetic of hybridization**. Panel A; homozygous wild type DNA, panel B: homozygous mutated DNA. Black bars correspond to the wild type probe (V617Fwt) and white bars correspond to mutated probe (V617Fmut). MFI: median fluorescent intensity.

Following the amplification of the JAK2V617F targeted region by PCR using control wild-type (CEPH, homozygous wild-type) and homozygous mutated (HEL cells, homozygous JAK2V617F/JAK2V617F mutant) genomic DNA, we determined the hybridization temperature profile of the wild-type and mutated probes coupled to their corresponding microspheres (Fig. 1). The hybridization temperature giving a good discrepancy between the wild-type and mutated probes was about 53°C. We then tested the effect of DNA concentration on the median fluorescence intensity (MFI) measured at that precise hybridization temperature (Fig. 2). The MFI was directly proportional to the DNA concentration for up to 5.0 μL of undiluted PCR product. No reduction of the MFI was observed at that concentration which sometimes occurred with longer PCR fragments (not shown). We tested the effect of time on the kinetic of hybridization for a maximum of 60 minutes (Fig. 3). When using 2.5 μL of PCR products, the reactions reached a plateau after 30 minutes of incubation at 53°C for both the wild and mutated types. No decrease in MFI was observed after 60 minutes of incubation.

### Standard curve

**Figure 4:**
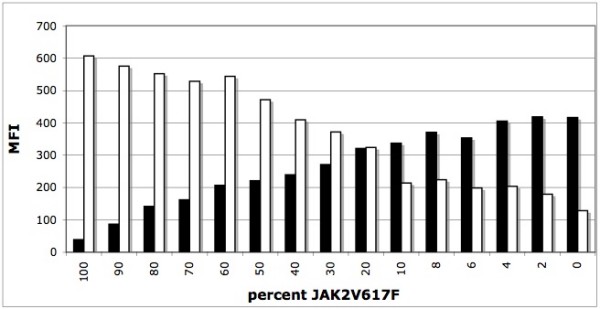
**Quantitative analysis of various ratios of wild type vs mutant alleles**. Various ratios of wild type and mutated alleles ranging from 0% up to 100% mutated allele were analyzed using direct hybridization. Black bars correspond to the wild type probe (V617Fwt) and white bars correspond to mutated probe (V617Fmut). MFI: median fluorescent intensity.

**Figure 5:**
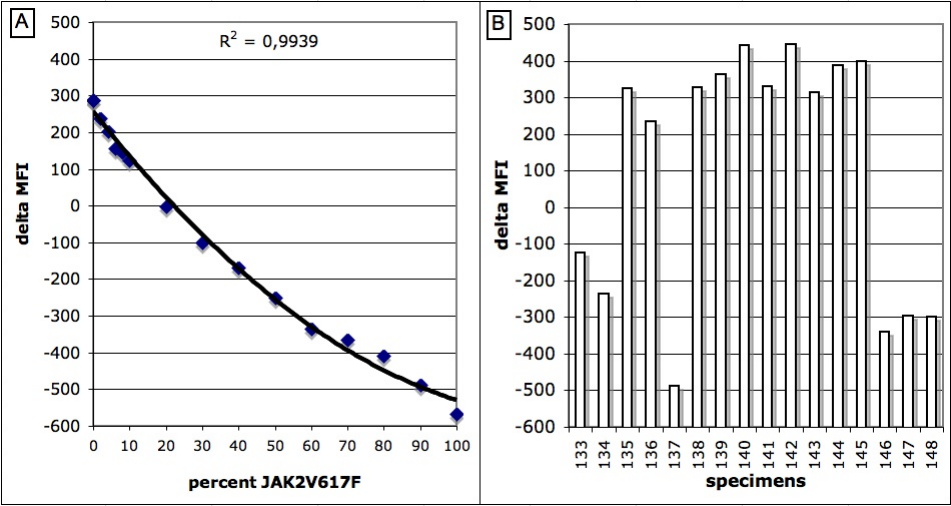
**Polynomial graphic representation of the standard curve (A) and quantitative detection of JAK2V617F from specimens (B)**. Typical representation of the delta MFI curves and bar graphs applied to the detection of JAK2V617F variant from specimens. The percentage of mutated alleles is determined by comparing the height of the bar graphs from specimens with the standard curve. The equation is Y = 0.0473X^2^-12.6X+256.

In order to generate the standard curve, we combined various amounts of wild-type and mutant PCR fragments ranging from 0, 2, 4, 6, 8, 10, 20, 30, 40, 50, 60, 70, 80, 90, up to 100% mutant (Fig. 4). The differences between the wild-type and mutated probes MFI values were plotted on a polynomial graphic (Fig. 5A). The R^2 ^values remained above 0.99 confirming the quantitative nature of our controls.

### Quantitative and qualitative analysis of JAK2V617F variants

The quantitative detection of the JAK2 variants was performed on specimens whole blood spotted onto FTA cards as described above. The MFI values obtained between the wild-type and mutated probes were subtracted from one another and the differences were used to generate a single bar graph (Fig. 5B). By comparing the delta MFI values obtained from the specimens with those of the standard curve (Fig. 5A), a percentage of mutant allele to wild-type was assessable with an estimated limit of detection of 2%. In order to increase the validity and sensitivity of the test, we combined a qualitative ASPE reaction to all specimens tested. All except one specimen negative for JAK2V617F by direct hybridization were negative by ASPE and all specimens positive for JAK2V617F by direct hybridization were positive by ASPE. In this typical example, specimens number 135 and 138 to 145 where given a negative results (absence of the V617F variant) whereas specimens number 133, 134, 136, 137, 146, 147 and 148 where given a positive results (presence of the V617F variant) with values of 35%, 47%, 3%, 90%, 62%, 55% and 55% mutant, respectively. Specimen 143 negative by DH and positive by ASPE was given a value of bellow 2% mutant.

With a 45 cycles PCR reaction, the ASPE assay had a higher sensitivity than the direct hybridization assay and could theoretically detect 0.1% of mutated cells in a wild-type background or 2 mutated alleles out of 2000 or 1 out of 1000 cells. The ASPE reaction included a 3'phosphate-blocked oligonucleotide corresponding to the wild-type allele [18]. This allowed a reduction of the background that sometime occurred from non-specific amplification reaction. A good and reproducible concordance was obtained between the direct hybridization reactions and the ASPE assays (r = 0.95, p < 0.05). Specimens wild type for the JAK2 alleles fluctuated slightly above the 0% standard control. The few that were positive by ASPE and negative by DH were assigned a value bellow 2% mutant.

## Discussion

Since the first reports in 2005 of the JAK2V617F variant, numerous genotyping techniques have been developed. Its detection has been included as a diagnostic criteria for PV by the World Health Organization. Sensitivities vary between 20% for DNA sequencing all the way down to 0.1% for ASPE. Some of them require exhaustive granulocyte DNA purification steps which is time consuming and sometimes difficult because monocytes, granulocytes and platelets may aggregate [19]. In addition, a recent study [20] suggests that platelets are better suited for JAK2V617F mutation screening since granulocytes analysis could lead to an underestimation of real carriers. This is also supported by recent studies that detected the JAK2V617F mutation in B- and T-lymphocytes [19] as well as in NK-cells [21,22] making the use of whole blood better suited then purified granulocytes, although this mutation has not been found in any lymphoid malignancy [6]. A recent study [23] comparing whole blood and purified blood granulocytes identified a good correlation between both sampling methods with allelic ratio ~15% higher on average for purified granulocytes. Using only the data from blood cell counts and correcting for the percentage of neutrophils, the percentage of mutated JAK2V617F fitted perfectly the values obtained from purified granulocytes.

In this study, we have developed and evaluated a quantitative assay for the detection of the JAK2V617F variant in the Jun activated kinase 2 gene using the Luminex platform. The Luminex technology offers two distinctive approaches, either the xTAG protocol or the xMAP protocol. The former requires a DNA extraction, end point PCR, an exo-sap treatment, an ASPE reaction followed by an hybridization at 37°C. The later requires a DNA extraction, an end point PCR followed by an hybridization at a specific temperature, in this case, at 53°C. Although the xMAP approach implies the predetermination of the optimal kinetic parameters, it requires less steps to perform. The xMAP technology requires a certain level of experience and technical skills, but is simple, sensitive and applicable at a reasonable cost. As starting material, we choose whole blood spotted onto FTA cards because of the genomic DNA stability, potential pathogen inactivation and ease of storage of the specimens. The methanol-water DNA extraction method proved to be simple and highly reproducible allowing multiple genotyping assays from a single 2 mm punch. The end point PCR amplification of the targeted region is straight forward and easily assessed for efficiency by agarose-gel electrophoresis.

We evaluated the possibility of using whole blood spotted onto FTA cards to detect the JAK2V617F genetic variant because of its numerous advantages including (1) the simplicity of collection of blood sample, (2) room temperature storage conditions without need for refrigeration, (3) pathogen inactivation that could be present in human blood, (4) fast purification of DNA and (5) overall low cost when compared to other DNA storage and purification methodologies. In addition, making use of the methanol-water extraction protocol decreases substantially the number of technical steps, time and costs associated with human genomic DNA isolation that is requires for PCR. In contrast, highly purified DNA is required to perform real time PCR making this approach more labor intensive and at increased costs. We believe that FTA cards are therefore a good and inexpensive way to store blood and test for the presence of the JAK2V617F mutation.

A recent report [11] on the concordance of various assays designed for the quantitative detection of JAK2V617F concluded that the allelic burden as defined by JAK2V617F/total JAK2 produced similar results with variable sensitivity using a common set of standards. It mentions the importance of using defined accurate standards for calibration. Our standards are composed of a mixture of two PCR products eliminating the effect of JAK2 gene copy number variation reported for the HEL cell line. The PCR fragment produced by CEPH DNA being 100% wild-type and the fragment produced by HEL genomic DNA being 100% mutant, a mixture composed of various amounts of both fragments generates standards that are simple to produce and can be directly compared to the PCR fragments from the submitted specimens. By comparison, later standards were composed of various dilutions of UKE-1 cells (homozygous mutant for JAK2V617F) and normal leukocytes (wild-type JAK2). The linear profile of the standard curve from the supplemental data does suggest that the values measured are accurate. We believe that both types of standards are accurate standards and the user may choose which is more convenient depending on their laboratory settings. We tested the 0%, 1%, 10%, 25%, 50%, 75% and 100% mutant control samples (kindly provided by S. Hermouet) and after appropriate dilutions, got values of 0%, 0%, 6%, 22%, 54%, 90% and 100%, respectively, which is a good concordance between both types of standards (data not shown). Our ASPE reaction gave 100% concordance when these controls were tested for the presence or absence of the mutated targeted sequence (not shown). The ASPE reaction did detect the 1% control sample whereas the Luminex DH assay did not which is in good agreement with our estimated limit of detection of 2%.

Patients with more than 50% of mutated JAK2 allele can harbor homozygote clones [22,24] and can carry wild-type, heterozygous and homozygous clones making questionable the true meaning of a specific percentage value. Some cells even carry multiple copies of the JAK2 gene originating from genetic duplication and recombination. The estimated percentages of JAK2V617F is therefore a value that must not be considered as an actual number of mutated cells compared to all nucleated cells in a sample but rather a variable number of affected cells. Patients with high percentage of JAK2V617F often carry the mutation in a homozygous state that has a direct effect on the true quantitative allele burden measured from whole blood cells.

Using our end point PCR allows the production of a single fragment for each specimen tested that should theoretically represent a constant final concentration assuming that the end point is reached a few cycles before the end of the amplification reactions. From the estimated 1000 genomes present in a single PCR reaction, we estimated that 35 cycles is sufficient to reach the end point assuming 100% amplification efficiency making 45 cycles sufficient for our assay. The five-fold dilution of the specimen PCR products prior to hybridization was implemented because of the ease of making the standard curve mixtures. This also allowed the use of the standard curve samples for many more genotyping assays. Our assay has the advantages of being simple, sensitive, and requires a single PCR reaction.

Although at very low frequency, other mutations have been reported in JAK2 exon 14 [25,26]. Those that are of particular interest for us are located within the probes targeted sequences and include C616Y and C618R. Theoretically, a compound heterozygous individual carrying the V617F variant on one allele and either C616Y or C618R on the other allele could not be detected unless different probes were used.

We used a standard end point PCR reaction in parallel with an ASPE reaction under identical amplification conditions to validate our assays. During the PCR reaction, both alleles are amplified using the same primers making the amplification efficiency identical for both alleles and consequently has little or no effect on the initial allelic ratios as reported elsewhere [13]. Since both PCR and ASPE reactions are ran under identical conditions and products visualized by agarose-gel electrophoresis, the results are easily comparable reinforcing the final diagnosis regarding the presence or absence of the JAK2V617F variant especially for patients with values bellow 2%.

Direct hybridization of the Luminex xMAP technology is a simple method taking advantage of the unique properties of DNA to form specific hybrids with its complementary sequences. Under specific hybridization conditions such as temperature, DNA concentration, salt concentration and time, one can discriminate two DNA sequences having a single nucleotide polymorphism. To our knowledge, this is the first report of a quantitative detection of a mutated allele linked to a human disease using this technology. We estimated our threshold of detection to be 2% which is similar to most highly sensitive detection technique reported to date. Furthermore, patients with lower than 2% allele burden of the mutated allele should not be considered positive as healthy patient do carry this mutation at very low copies as previously reported [14]. Increased sensitivity could also lead to false positives.

Other mutations relevant to hematopoietic and myeloid malignancies have been reported [26] and may be detectable by multiplexed PCR followed by our Luminex assay, although the quantitative nature of multiplexing and using direct hybridization is presently under investigation. The next step would be to add other related mutations to evaluate the possibility of multiplexing our quantitative assay as other mutations in either exon 12, 13 and 15 of JAK2 [27,28] or other genes [26] linked to MPN have recently been reported.

## Conclusion

Direct hybridization assay using the Luminex xMAP technology allows sensitive quantification of JAK2V617F from blood spots. It is simple and can be easily performed to support the diagnosis or clinical management of myeloproliferative diseases.

## Competing interests

The authors declare that they have no competing interests.

## Authors' contributions

The original idea was proposed and specimens were provided by RS. FWP conceived the assay design, validation and implementation. FWP and DG drafted the manuscript. All authors read and approved the final manuscript.

## Pre-publication history

The pre-publication history for this paper can be accessed here:

http://www.biomedcentral.com/1471-2350/11/54/prepub
